# Use of an Autologous Liver Round Ligament Flap Zeros Postoperative Bile Leak after Curative Resection of Hilar Cholangiocarcinoma

**DOI:** 10.1371/journal.pone.0125977

**Published:** 2015-05-04

**Authors:** Da-Xin Sun, Xiao-Dong Tan, Feng Gao, Jin Xu, Dong-Xu Cui, Xian-Wei Dai

**Affiliations:** Department of General Surgery, Shengjing Hospital of China Medical University, Shenyang, Liaoning Province, 110000, China; University Hospital Oldenburg, GERMANY

## Abstract

**Background:**

Postoperative bile leak is a major surgical morbidity after curative resection with hepaticojejunostomy for hilar cholangiocarcinoma, especially in Bismuth-Corlette types III and IV. This retrospective study assessed the effectiveness and safety of an autologous hepatic round ligament flap (AHRLF) for reducing bile leak after hilar hepaticojejunostomy.

**Methods:**

Nine type III and IV hilar cholangiocarcinoma patients were consecutively hospitalized for elective perihilar partial hepatectomy with hilar hepaticojejunostomy using an AHRLF between October 2009 and September 2013. The AHRLF was harvested to reinforce the perihilar hepaticojejunostomy. Main outcome measures included operative time, blood loss, postoperative recovery times, morbidity, bile leak, R0 resection rate, and overall survival.

**Results:**

All patients underwent uneventful R0 resection with hilar hepaticojejunostomy. No patient experienced postoperative bile leak.

**Conclusions:**

The AHRLF was associated with lack of bile leak after curative perihilar hepatectomy with hepaticojejunostomy for hilar cholangiocarcinoma, without compromising oncologic safety, and is recommended in selected patients.

## Introduction

Hilar cholangiocarcinoma, also called Klatskin tumor, is a biliary tract cancer that mainly involves the common hepatic duct, the right and left hepatic bile ducts, and their confluence [1]. This cancer is infrequently encountered in clinical practice, with an overall incidence of 1.2 per 100000 persons, and accounts for approximately 2% of all cancer cases and only 10% of all biliary tract cancers [2]. Although the etiology of hilar cholangiocarcinoma remains unclear, aging (over 65 years) is a known risk factor, and the associated chronic biliary epithelial injury predisposes an individual to carcinogenesis [3]. Hilar cholangiocarcinoma is an occult malignant disease, and in an early stage tends to be asymptomatic. With disease progression, it can result in obstructive jaundice [4]. Often, by the time the patient seeks medical treatment the disease is unresectable and the prognosis of hilar cholangiocarcinoma is discouragingly poor, even compared with that of hepatocellular carcinoma [5].

Curative hilar hepatectomy with concomitant biliary tract reconstruction remains the definitive mainstay treatment, offering the possibility of favorable long-term survival [6]. Liver transplantation [7] and adjuvant therapies [8] may be attempted in some cases. However, surgical treatment of hilar cholangiocarcinoma is technically challenging and subject to a high risk of morbidity, especially in jaundiced patients [9]. Bile leak is a common procedure-related complication after perihilar hepatectomy, which is more frequently observed in cases of Bismuth-Corlette types III and IV requiring major perihilar hepatectomy [10] in which the presence and retraction of multiple intrahepatic bile duct stumps might complicate subsequent hepaticojejunostomy. Wahab et al. [11] reported a bile leak frequency of 23.2% in 73 Egyptian patients undergoing curative local or extended perihilar hepatectomy. Bile leak may result in an imbalance of water and electrolytes, surgical site infection, and liver failure especially in patients with impaired liver function reserve, as well as life-threatening bile peritonitis [12]. Complex bile leak requires endoscopic intervention or even re-laparotomy.

Autologous tissue or synthetic biomaterial mesh is widely used in current surgical practice for prophylaxis and treatment of a variety of anastomotic leaks, mainly in biliary [13,14], gastrointestinal [15], cardiothoracic, urological, and gynecological surgeries. Hepatic round ligament (HRL), also called the *ligamentum teres hepatis*, is the remnant of the fetal umbilical vein and is connected to the falciform ligament. This ligament is well vascularized and extensible [16], and can therefore be harvested for enhancing venous- [17] or pancreatico-enterostomy[18].

The present retrospective study evaluated the effectiveness and safety of using an autologous HRL flap (AHRLF) for reducing bile leak originating from hepaticojejunostomy in patients receiving curative resection of hilar cholangiocarcinoma.

## Material and Methods

### Patients

All patients voluntarily gave informed consent in writing prior to surgery. The experimental protocol was established, according to the ethical guidelines of the Helsinki Declaration and was approved by the Human Ethics Committee of Shengjing Hospital of China Medical University, China.

Nine patients with suspected hilar cholangiocarcinoma were consecutively hospitalized for curative resection with concomitant hilar cholangiocarcinoma at the Department of General Surgery, Shengjing Hospital of China Medical University, between October 2009 and September 2013. The indications for curative perihilar hepatectomy with hepaticojejunostomy using the AHRLF were: (1) a suspected hilar cholangiocarcinoma classified as Bismuth-Corlette types III and IV, namely involving the right or left hepatic ducts, or both, and the convergence of the hepatic ducts [19]; (2) hilar bile duct tumor determined on medical imaging to be resectable by local perihilar hepatectomy; and (3) the presence of multiple intrahepatic bile duct openings, refractory to conventional hepaticojejunostomy. The contraindications were: (a) requiring extended hemihepatectomy for a clean resection margin, determined by intraoperative frozen biopsy; (b) complicating serious impairment of cardiopulmonary or hepatorenal function, coagulopathy, or local or systemic infection; or (c) intrahepatic or intraperitoneal metastasis.

**Fig 1:**
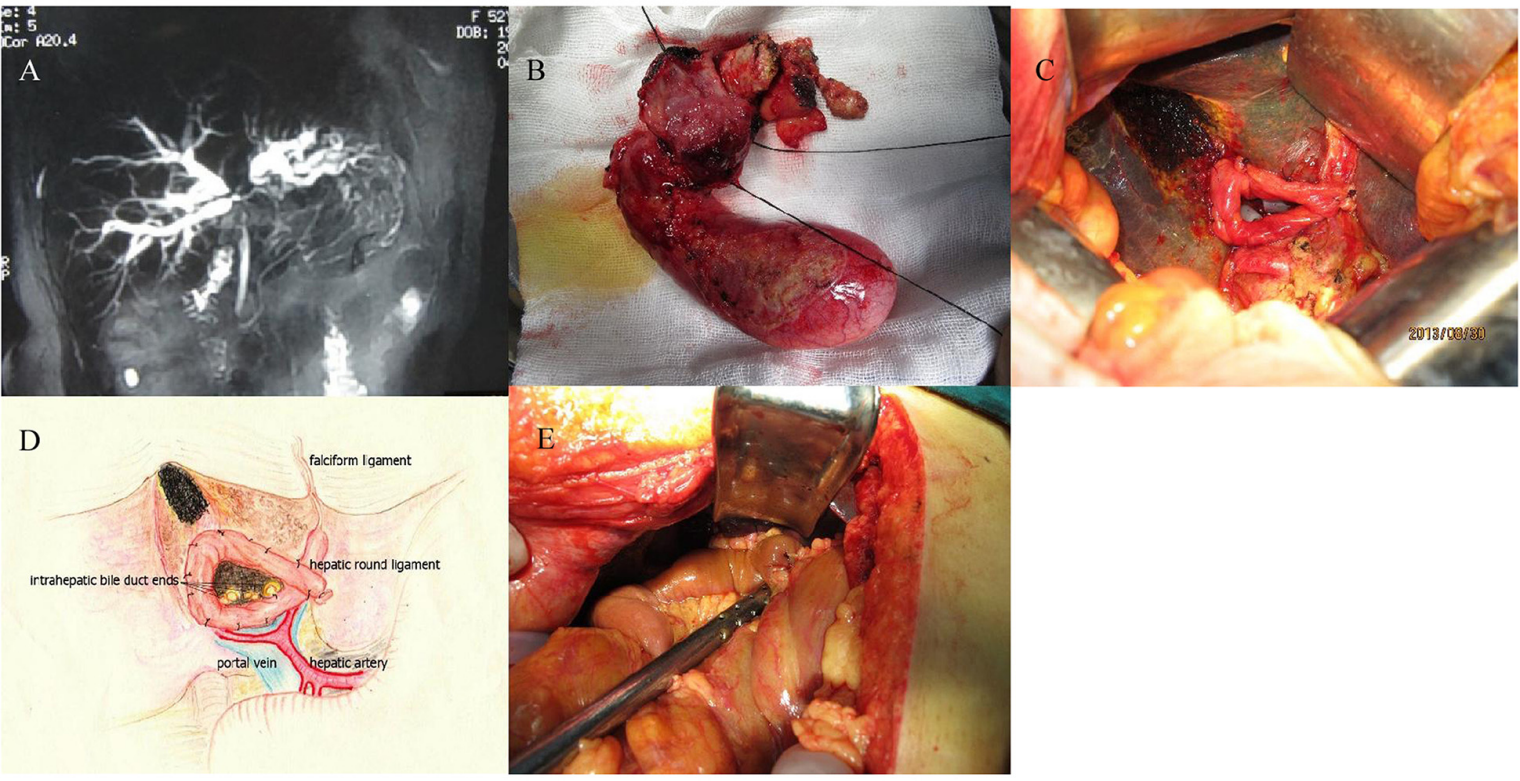
Preoperative imaging examination of hilar tumor and key surgical procedures. (A) Representative appearance on magnetic resonance cholangiopancreatography; (B) *en bloc* resection of the hilar tumor; (C) appearance of multiple intrahepatic bile duct openings on the hilar stump; (D) circumferential enhancement of the hilar stump with the AHRLF; and (E) anastomosis of the Roux-en-Y hepaticojejunostomy with the AHRLF.

Preoperative medical imaging examinations, including hepatobiliary ultrasonography and computed tomography scan and magnetic resonance cholangiopancreatography, were performed to locate and delineate the hilar tumor (Fig 1A.). Preoperative conditioning treatment included symptomatic hepatoprotective therapy such as medication with vitamin K_1_, and biliary decompression with percutaneous transhepatic cholangial drainage for jaundiced patients (total bilirubin > 400 μmol/L).

### Surgical procedures

All operations were performed by an assigned surgical team led by the corresponding author (XWD), comprising attending and resident surgeons, anesthesiologists, clinical pathologists, radiologists, surgical nurses, and research staff.

The patient was placed in the supine position for routine laparotomy. Under general anesthesia with endotracheal intubation, an upper midline incision with bilateral subcostal extensions was performed.

### Harvest of hepatic round ligament

After confirming the resectability of the tumor and the absence of any signs of intra- and extrahepatic metastases, the HRL was meticulously dissected using electrocauterization, starting from the falciform ligament and towards the umbilicus, and transected and harvested, as long as possible, with the pedicle preserved. A frozen biopsy was performed to exclude possible tumor contamination.

### Perihilar hepatectomy

For hilar hepatectomy, the hepatoduodenal ligament was thoroughly skeletonized as follows. The right gastric vessels were transected; the station 8 lymph nodes were dissected; and the proper hepatic artery was dissected towards the hilum. The station 12p and 12a lymph nodes were dissected, and then the station 12b and 13 lymph nodes were dissected. The common bile duct was transected at the level of the superior margin of the pancreas. The lower common bile duct lumen was thoroughly flushed and closed with uninterrupted non-invasive sutures. The upper common bile duct stump was retracted upwards for hilar dissection with the preservation of the portal vein, and the right hepatic artery located posterior to the common hepatic duct was cautiously dissected and preserved.

Cholecystectomy was performed in a retrograde manner, towards Calot’s triangle. Partial hepatectomy was performed using electrocauterization 2 cm above the transverse groove of the hepatic hilum. The intrahepatic vessels were transected and ligated to minimize blood loss, and the left and right hepatic ducts were exposed. The dilated hepatic ducts were bluntly dissected and transected, and the hilar tumor located at the convergence of the hepatic ducts was removed *en bloc* (Fig 1B.). A frozen section biopsy was performed to confirm the radicality of the resection.

### Management of the hilar stump

After removal of the hilar tumor, the hilar stump exhibited multiple intrahepatic bile duct openings, including those draining the right anterior, right posterior, caudate, left anterior and left posterior lobes from right to left (Fig 1C.). The hilar stump was confined by the hepatic stump on the top, and the inferior or lateral margin of the intrahepatic bile ducts and the bifurcation of the right and left hepatic arteries on the bottom. Excessive adipose tissue was excised to revise the HRL.

### Hepaticojejunostomy

A 40-cm-long Roux-en-Y jejunal limb was harvested and brought to the hepatic hilum through an avascular opening in the transverse colon mesentery. A 2-cm jejunostomy was created on the anti-mesenteric margin, 3 cm distant to the closed end of the jejunal loop. The AHRLF was circumferentially sutured to the Glisson’s capsules on the hilar stump using 4–0 absorbable sutures (Fig 1D.). The jejunal loop was anastomosed with the AHRLF ring using interrupted 4–0 absorbable sutures, in a full-thickness manner (Fig 1E.

### Postoperative care and follow-up

A peritoneal drain was placed to monitor the peritoneal drainage with respect to volume, content, and color. Bile leak was defined bilirubin concentration in the drain fluid at least 3 times the serum bilirubin concentration on or after postoperative day 3 [20]. Postoperative care included nutritional support, liver protection, and antimicrobial prophylaxis. No adjuvant chemotherapy or radiation therapy was given. All patients were followed up using routine hematology, liver biochemistry, and upper abdominal computed tomography scan at outpatient clinics 3, 6, and 12 months after the surgery, and at 6-month intervals afterwards.

## Results

### Patient characteristics

Nine Bismuth-Corlette type III or IV patients were treated using perihilar hepatectomy with hepaticojejunostomy enhanced by the AHRLF (Table 1).

**Table 1:** Demographic and clinical characteristics of hilar cholangiocarcinoma patients (n = 9).^a^

Age, y		64 (58−73)
Gender, male:female		5:4
Body mass index, kg/m^2^		27.6 (23.4−30.2)
History of disease, month		1.2 (0.5−2)
Liver function	Alanine transaminase, U/L	99 (54−179)
	Aspartate aminotransferase, U/L	72 (43−105)
	Total bilirubin, μmol/L	232 (87−420)
	Conjugated bilirubin, μmol/L	187 (62−310)
	Unconjugated bilirubin, μmol/L	45 (25−110)
	Serum albumin, g/L	36 (32.3−39.5)
	Carbohydrate antigen 19–9, U/mL	302 (58−1000)
Underlying liver conditions, n (%)	Chronic hepatitis	11.1
	Comorbidities, n (%) ^b^	33.3

^a^ Median (range), unless noted otherwise;

^b^ surgical and medical

### Operative results

In all patients, the HRL was successfully harvested without tumor contamination. The perihilar hepatectomy with hepaticojejunostomy was uneventfully completed in all patients, at a mean operative time of 4.3 hours. The mean bleeding volume was 180 mL, and no patient required blood transfusion (Table 2).

**Table 2:** Operative and postoperative recovery data.*

Overall operative time, h		4.3 (3.5−5.5)
Operative bleeding, mL		180 (100−250)
Specimen size, cm		2.6 (2−3.5)
Positive/overall lymph nodes resected, n		2/18 (0/16−3/19)
Postoperative recovery, d	To restart off-bed activities	3.6 (2.5−5)
	To resume oral intake	3.3 (3−4)
	To resume bowel movement	3.3 (3−4)
	Discharge from hospital	14.3 (12−18)

* Median (range)

### Postoperative recovery and complications

All patients had an uneventful postoperative course, except for one patient experiencing pyrexia, which was determined to be septic cholangitis and resolved by bacterial culture-based antimicrobial treatment. None of the patients suffered hemobilia, bile leak (presence of bile juice in the drainage), or acute liver failure. Patients resumed off-bed activities, bowel movements, and oral intake 3 days after surgery. The mean duration of postoperative hospitalization was 14.3 days (range, 12−18), and there was no in-hospital mortality (Table 2).

### Oncologic and follow-up results

By histology, all 9 patients received R0 resection. Four, 3, and 2 patients had, respectively, well, moderate, and poorly differentiated bile duct adenocarcinoma. All patients were followed as scheduled for 3 to 6 months, and overall survival was 18 to 34 months (median, 25 months).

## Discussion

Perihilar hepatectomy with hepaticojejunostomy for curative treatment of hilar cholangiocarcinoma is a high-risk hepatobiliary operation subject to substantial morbidities and mortality, especially when major hepatectomy is performed on patients with serious cholestasis, such as in cases of Bismuth-Corlette types III and IV. The overall morbidity is reported to range from 40 to 80% [21], and the mortality rate is up to 10% [22]. Long operative time and excessive blood loss have been shown to be associated with a significantly higher morbidity [20]. Our results achieved a minimal morbidity and zero mortality, although it is noted that only local perihilar hepatectomy was performed on our patients.

Biliary reconstruction after perihilar hepatectomy is relatively complex, due to the presence and retraction of multiple intrahepatic bile ducts on the stump. Bile leak is a less common (2.6–12%) but major surgical safety concern after hepaticojejunostomy, and its occurrence is dependent on a number of factors, both patient and surgical [11]. Poor baseline liver function and underlying cholestasis or cholangitis predispose patients to a higher risk of postoperative bile leak [23]. Preoperative biliary decompression is likely to improve liver function, cholestasis, and cholangitis and contribute to reduction of bile leak risk [24]. Extended transection involving liver segments 1 and 4 has also been reported to increase risk of bile leak [25], while use of fibrin glue to control liver stump oozing may reduce bile leak [26]. Moreover, a wide-interval biliary enteric anastomosis is thought to minimize biliary anastomotic stricture and bile leak. Nevertheless, hepaticojejunostomy after curative resection of hilar cholangiocarcinoma, especially if involving the convergence of hepatic ducts, is generally believed to be more prone to bile leak compared with benign bile duct diseases [27].

The HRL has been historically used for prophylaxis and repair of gastrointestinal perforation or bleeding. Costalat et al. [28] reported successful repair of gastric perforation using an HRL flap in patients with peptic ulcer. Iannitti and his colleagues [29] harvested the HRL to circumferentially secure the duct-to-mucosa pancreaticojejunostomy. There was no incidence of pancreatic fistula, which was attributed to the vascularizing effect of the HRL. The absence of pancreatic fistula further contributed to fewer surgical morbidities and expedited postoperative recovery. Nakatsuka et al.[30] also reported that suture of the HRL to the gastroduodenal stump could minimize bleeding from the proper and common hepatic arteries after pancreaticoduodenectomy. Moreover, Zhang and his colleagues [31] reconstructed the bile duct defect after choledochectomy using the HRL for a type IIa hilar cholangiocarcinoma in an elderly patient, and subsequently anastomosed the posterior margin of the jejunal limb with the HRL for Roux-en-Y hepaticojejunostomy. In summary, the potential clinical benefits of using the AHRLF for hepaticojejunostomy include protecting the hepaticojejunostomy anastomosis blood supply; allowing a radical resection of the bile duct as proximally as needed to ensure an oncologically negative resection margin (by offering a highly flexible bile ductoplasty); and minimizing incidental injury of the portal vein bifurcation and consequent portoenteric fistula. In addition, our study shows that enhancing hepaticojejunostomy with the AHRLF can result in zero incidence of bile leak and minimal overall and biliary complications.

We acknowledge that this study is limited in that the sample size was very small, because hilar cholangiocarcinoma is encountered only occasionally. Secondly, there was no control group of patients for comparison, undergoing the procedure without the AHRL. Moreover, the oncologic safety of the AHRLF remains unknown, although a long-term follow-up study is ongoing at our institute.

In conclusion, use of the AHRLF is feasible and effective in reducing the occurrence of bile leak after perihilar hepatectomy with hepaticojejunostomy for curative treatment of hilar cholangiocarcinoma, in selected patients with a localized disease not requiring extended resection. Incorporation of AHRLF also helped simplify the operative procedure, especially when anastomosing the jejunal loop with the retracted bile duct openings on the stump. HRL harvest is normally free of tumor contamination and does not compromise oncologic safety. Large-scale randomized controlled studies are required to confirm that this technique minimizes bile leak and other biliary complications, and provides satisfactory oncologic adequacy and survival outcome. A prospective study is ongoing at our institute to evaluate the oncologic effectiveness and safety of perihilar hepatectomy and extended hepatectomy for type III/IV hilar cholangiocarcinoma, with a clean resection margin as a major stratifying factor.
